# Genetic features of Sri Lankan elephant, *Elephas maximus maximus* Linnaeus revealed by high throughput sequencing of mitogenome and ddRAD-seq

**DOI:** 10.1371/journal.pone.0285572

**Published:** 2023-06-13

**Authors:** M. G. C. Sooriyabandara, J. M. S. M. Jayasundara, M. S. L. R. P. Marasinghe, H. A. B. M. Hathurusinghe, A. U. Bandaranayake, K. A. N. C. Jayawardane, R. M. R. Nilanthi, R. C. Rajapakse, P. C. G. Bandaranayake

**Affiliations:** 1 Department of Wildlife Conservation, Battaramulla, Sri Lanka; 2 Agricultural Biotechnology Centre, Faculty of Agriculture, University of Peradeniya, Peradeniya, Sri Lanka; 3 Department of Computer Engineering, Faculty of Engineering, University of Peradeniya, Peradeniya, Sri Lanka; 4 Department of National Zoological Gardens, Anagarika Dharmapala Mawatha, Dehiwala, Sri Lanka; State Museum of Natural History, GERMANY

## Abstract

*Elephas maximus maximus* Linnaeus, the Sri Lankan subspecies is the largest and the darkest among Asian elephants. Patches of depigmented areas with no skin color on the ears, face, trunk, and belly morphologically differentiate it from the others. The elephant population in Sri Lanka is now limited to smaller areas and protected under Sri Lankan law. Despite its ecological and evolutionary importance, the relationship between Sri Lankan elephants and their phylogenetic position among Asian elephants remains controversial. While identifying genetic diversity is the key to any conservation and management strategies, limited data is currently available. To address such issues, we analyzed 24 elephants with known parental lineages with high throughput ddRAD-seq. The mitogenome suggested the coalescence time of the Sri Lankan elephant at ~0.2 million years, and sister to Myanmar elephants supporting the hypothesis of the movement of elephants in Eurasia. The ddRAD-seq approach identified 50,490 genome-wide SNPs among Sri Lankan elephants. The genetic diversity within Sri Lankan elephants assessed with identified SNPs suggests a geographical differentiation resulting in three main clusters; north-eastern, mid-latitude, and southern regions. Interestingly, though it was believed that elephants from the Sinharaja rainforest are of an isolated population, the ddRAD-based genetic analysis clustered it with the north-eastern elephants. The effect of habitat fragmentation on genetic diversity could be further assessed with more samples with specific SNPs identified in the current study.

## Introduction

The Asian elephant (*Elephas maximus* Linnaeus, 1758) is an endangered species [1], and reported throughout South-East Asia, including Sumatra, Java, Borneo, and China, as well as West Asia along the Persian coast and the Indian subcontinent [2, 3]. Four subspecies have been recognized from different geographical regions [2, 4] and differentiated by their size and the variation in pigmentation pattern [5–7]. They are the Indian elephant, *Elephas maximus indicus* Cuvier from Asian mainland/India (1798) [8], Sumatran elephant, *Elephas maximus sumatranus* Temminck (1847), Sri Lankan elephant, *Elephas maximus maximus* Linnaeus (1758), and later the Borneo pygmy elephant, *Elephas maximus borneensis* (1970) was identified [6, 9, 10]. *Elephas maximus maximus* has the largest body size and the darkest skin color compared with other subspecies, while the skin color of *E*. *m*. *indicus*, with smaller patches of depigmentation, is darker than that of *E*. *m*. *sumatranus* [7]. A lower level of heterozygosity was identified among the Asian elephants [11], and no evidence of nuclear gene flow and genetic divergence splitting the Asian elephants into four subspecies. Early studies depicted phylogeographical differences between the mainland and Sri Lankan elephant populations [9, 12–14].

The estimation of mitochondrial displacement loop (mt D-loop) sequence variation is currently the most common genetic approach for speciation and population genetics [5, 15]. It is recorded to have existence of two divergent lineages of mt D-loop haplotypes in Asian elephants [13], as α and β clades, with approximately 3% sequence divergence, and they co-exist across the Asian continent [9, 12, 13, 16, 17]. The α haplogroup is abundant on the Asian mainland, with only a few inhabitants in Sri Lanka, while the β haplogroup is extensively dispersed in Sumatra and Sri Lanka [5, 14, 16]. The β haplogroup consists of two sub-clades as β1 and β2, distributed primarily in Sri Lanka and South and Central India, with a few populations in Myanmar. Similarly, admixtures of α and β1 populations have been observed in Thailand as well as Myanmar. The β2 clade is purely distributed in Sumatra. Other than the non-coding D-loop region, previous work is available on cytochrome b (Cyt b) [13] and NADH dehydrogenase subunit 5 (ND5) [6].

Asian elephants are forest animals that are considered endangered due to widespread and intense elephant-human conflict [2, 18]. Approximately only 50,000 Asian elephants remain in fragmented populations across 13 range states in Asia [2]. It has drawn immediate attention to and necessity of elephant management activities and conservation practices. Sri Lanka is home to the second largest population of Asian elephants, mainly confined to the lowlands in the dry zone in the north, south, east, north-western, north-central, and southeastern forest reserves of the country [13]. However, the Sri Lankan elephant has been understudied compared to other Asian elephants. Further, a recent survey recorded geographically distinct small remnant populations in the Sinharaja Rainforest and Peak Wilderness reserves in the wet zone [19]. However, it is believed all the other Sri Lankan elephants are entirely contiguous and are not geographically subdivided [19]. Nevertheless, scientists believe that dry zone elephants belong to a contiguous population despite small groups found in a few areas [19]. However, the morphological diversity of elephants observed in different regions and a mitochondrial genome-based analysis of northern, middle, and southern areas of Sri Lanka [12, 14] do not support that idea. Therefore, a rigorous genetic study is needed to ascertain the distribution and fragmentation of the elephant population in Sri Lanka.

The majority of work on Sri Lankan elephants in the public domain focused on behaviour, ecology, and conservation [12, 19, 20–25]. Few phylogeny and population genetics studies based on morphology and molecular traits have also been reported since the early 1990 [14, 26, 27]. The molecular techniques included mitochondrial markers, protein electrophoretic [11], and microsatellite loci [28, 29]. However, such studies have employed a small number of microsatellites and mtDNA markers and are insufficient to assess the population structure and admixture patterns in detail. Moreover, our recent work on 24 previously utilized SSR loci suggested that those are not specific to the *E*. *m*. *maximus* [30]. Therefore, the identification of a suitable set of markers is an urgent need for both conservation and management of *E*. *m*. *maximus* specifically and elephants in general. To lay the foundation, here we describe the first *de novo* assembly and annotation of the mitogenome of the Sri Lankan elephant. We generated the first genome-wide SNP dataset for Asian elephants with ddRAD sequencing data from 24 diverse individuals of *E*. *m*. *maximus*. We also assessed the evolutionary relationships of Asian elephants using available sequencing data.

## Materials and methods

### Ethical clearance

The study was conducted under the research permit number: WL/3/2/2017/1 issued by the Research Committee of the Department of Wildlife Conservation, Sri Lanka (DWC). We followed relevant guidelines and regulations for animal research approved by the committee. The DNA samples for sequencing were exported under CITES (Convention on International Trade in Endangered Species of Wild Fauna and Flora) permit (Security Stamp 1888968) obtained from the DWC.

### Sample collection and DNA extraction

**Fig 1:**
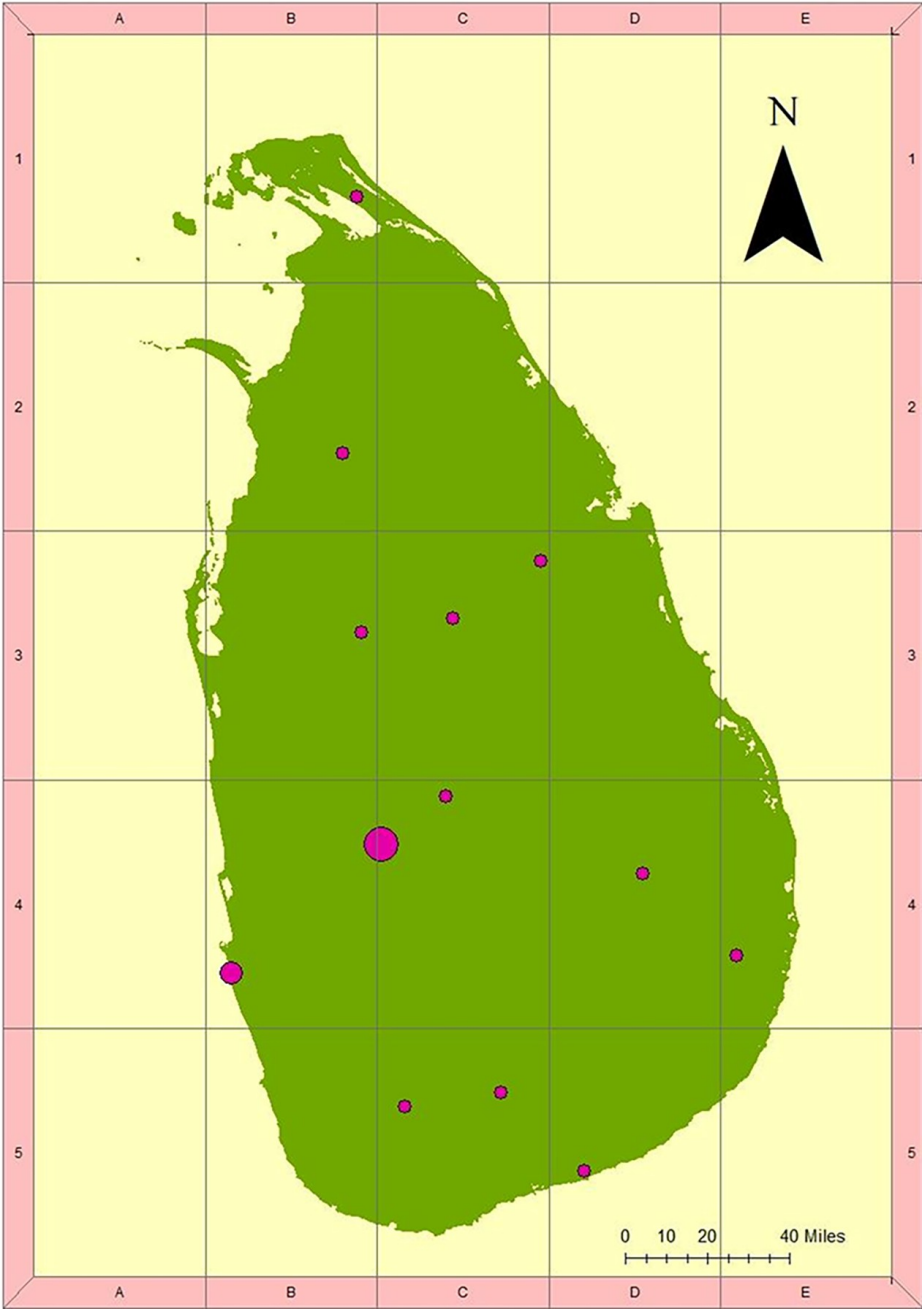
Distribution of elephant sampling localities throughout Sri Lanka. The circles are proportional to the ratio of elephants sampled. The number and location of samples used for the ddRAD analysis are given in S1 Table.

Twenty-four fresh whole blood samples were collected directly from both domestic and wild elephants with known origin and morphology (S1 Table, S1 Fig). Animals included both the sexes and tuskers (S1 Table). All the elephants are unrelated and born in widely distributed areas (Fig 1). Blood was collected from elephants after getting written informed consent from the Pinnawala Elephant Orphanage, following the ethical clearance guidelines.

Elephant blood DNA was extracted using the Wizard Genomic DNA Purification kit (Promega, A1120) following the manufacturers’ guidelines. DNA samples were quantified using the Nanodrop 2000 Spectrophotometer (Thermo Scientific) and assessed the quality with Nanodrop readings and running on a 1% agarose gel. The DNA samples with 230/280 and 260/280 values of about 1.80–2.00 and a clear single band on agarose gel were processed. The total DNA amount was adjusted to about 1–1.5 ug. For ddRAD sequencing, all 24 samples were sent to Admera Health Biopharma Services, New Jersey, USA.

### Genomic library preparation

Isolated genomic DNA was quantified with Qubit 2.0 DNA HS Assay (ThermoFisher, Massachusetts, USA) and quality assessed by Tapestation genomic DNA Assay (Agilent Technologies, California, USA). The total DNA concentration was adjusted to about 1.00 ug and the quality values of DNA Integrity Number (DIN) above 9 were considered satisfactory. The sequencing libraries were prepared using the KAPA Hyper Prep kit (Roche, Basel, Switzerland) per the manufacturer’s recommendations with Illumina® 8-nt dual-indices. The quantity of the final libraries was assessed by Qubit 2.0 (ThermoFisher, Massachusetts, USA), and quality was assessed by TapeStation D1000 ScreenTape (Agilent Technologies Inc., California, USA) where electopherograms and DIN values were taken as parameters. Libraries were sequenced on an Illumina HiSeq (Illumina, California, USA) with a read length configuration of 150 PE for 600 M PE reads per sample (300 M in each direction).

### *De novo* assembly and annotation of *Elephas maximus maximus* mitochondrial genome

We used the Mitoz v2.3 [31] program for *de novo* reconstruction, and annotation of *E*. *m*. *maximus* mitochondrial genome. According to the instruction given in Mitoz, 3-5GB of data is sufficient to completely assemble a mitochondrial genome, and we used BBMap *reformat*.*sh* [32] tool to extract ~5GB amount of reads (45,499,065 read pairs) from the whole genome dataset. However, the *de novo* assembler wasn’t successful in reconstructing the D-loop region of the circular genome. This region was still not resolved after using the multi-kmer mode recommended in Mitoz. The reason could be the inability of short reads to resolve and assemble such complex and repetitive regions [33]. Therefore, the whole-genome sequencing reads were mapped to the D-loop region of *Elephas maximus* mitochondrial genome (NC_005129) [34] using BWA MEM v0.7.17 [35], and the generated consensus sequence was merged into the assembly using the Geneious Prime 2020.1.2 (http://www.geneious.com/) software. After the manual curation of annotations, OrganellarGenomeDRAW v1.3.1 [36] was used to visualize the assembled genome.

### MtDNA divergence dating and nucleotide diversity analysis

We downloaded seven Asian elephant whole-genome datasets and two African elephant whole-genome datasets from NCBI SRA (National Center for Biotechnology Information—Sequence Read Archive). All whole-genome sequencing datasets (Table 1) were mapped to the *Elephas maximus* mitochondrial genome (NC_005129) [34] using BWA MEM (v0.7.17) [35]. Geneious software-generated consensus sequences and MAFFT aligner (v.7.450) [37] with its default configuration performed multiple sequence alignment. We also included *Mammuthus primigenius* (NC_007596; origin—Berelekh, Yakutia) [38], *Elephas maximus* (NC_005129; origin–Burma) [34] from NCBI repository, and *E*. *m*. *maximus* mitochondrial genome (this study) for the sequence alignment. The mtDNA D-loop region was excluded from the alignment.

**Table 1:** Whole-genome sequencing libraries used in the mitochondrial divergence dating.

Specimen	Species	Sex	Geographic Location	Source
*Moola* [39]	*Elephas maximus*	F	Myanmar	ERR2260498
*Chendra* [39]	*Elephas maximus*	F	Borneo [Malaysia]	ERR2260499
*Icky* [40]	*Elephas maximus*	F	Myanmar	SRR11577048
*Parvathy* [41]	*Elephas maximus*	F	India	SRR2008170
*Asha* [41]	*Elephas maximus*	F	India	SRR2009586
*Uno* [41]	*Elephas maximus*	F	Assam, India	SRR2012205
				SRR2012206
				SRR2012207
*Jayaprakash* [42]	*Elephas maximus*	M	Karnataka, India	SRR2912975
*Kadol*	*Elephas maximus*	M	Pinnawala, Sri Lanka	This study
*Watoto* [39]	*Loxodonta africana*	F	Kenya	ERR2260496
*Swazi* [39]	*Loxodonta africana*	F	Kruger National Park, South Africa	ERR2260497

For the nucleotide diversity analysis, we used the multiple sequence alignment of all the Asian elephant consensus mitogenomes and the *E*. *m*. *maximus* mitochondrial genome (this study) using the MAFFT aligner (v.7.450) [37] with its default configuration. This alignment was used to test the nucleotide diversity within the Asian elephant species by using the DnaSP v6.12.03 [43] We calculated Nucleotide variability (Pi) using the sliding window method with a window length of 200 bp and step size of 50 bp.

The divergence time of the elephant specimen was estimated using the BEAST v2.6.5 software package with the fossil calibration date of *E*. *maximus* and *M*. *primigenius* as a normal distribution with a mean of 6.01 Mya [44, 45]. The substitution rate was determined using the HKY model with a relaxed lognormal clock while the divergence was determined using the Yule node calibration technique. The posterior distributions were obtained by Markov chain Monte Carlo (MCMC) sampling with 10,000,000 steps while logging every 10,000 steps. The output was examined using Tracer v1.7.2 [46], and the maximum credibility tree was obtained using TreeAnnotator v2.6.4 (from BEAST package) after discarding 10% MCMC steps as postburn-in and visualized with FigTree v1.4.4 [47].

### Optimizing ddRAD approach for Sri Lankan elephants

#### ddRAD seq library preparation

Isolated genomic DNA was quantified with Qubit 2.0 DNA HS Assay (ThermoFisher, Massachusetts, USA) and quality assessed by Tapestation genomic DNA Assay (Agilent Technologies, California, USA The adapters were selected based on the restriction combinations. After the double digestion of the restriction enzymes, the samples were pooled before size selection. The target DNA fragments were enriched to prepare libraries by PCR amplification. The PCR products were purified, the final libraries quantity was assessed by Qubit 2.0 (ThermoFisher, Massachusetts, USA) and quality was assessed by TapeStation D1000 ScreenTape (Agilent Technologies Inc., California, USA). Based on QC values, Illumina 8-nt dual-indices were used and pooled equimolar libraries. Illumina HiSeq sequencing was performed (Illumina, California, USA), with a read length configuration of 150 PE for 6.6 M PE reads per sample (3.3M in each direction).

#### *In silico* enzyme selection and fragment size selection

We tested 16 different enzyme combinations (S2 Table) *in silico* to digest the genome using the tool FRAGMATIC [48]. This tool estimates the number of cut sites for given fragment sizes. The restriction enzyme combinations included four base + eight base cutters, six base + four base cutters, and six base + six base cutters. The four base + eight base cutting enzyme pairs, and six base + four base enzyme pairs usually produce a higher number of fragments, which makes it difficult to control errors, and requires deep sequencing depth. Meanwhile, the six base + six base enzyme pairs generally produced 20000–40000 fragments, which makes it easy to obtain a sufficient number of fragments without sequencing to a higher sequencing depth. We estimated the number of fragments in the range of 200–400 bp for each enzyme combination using the available genomes, *E*. *maximus* [36], *Loxodonta africana* LoxAfr3 (Ensembl 101 release), *Loxodonta africana* LoxAfr4 [39] and *E*. *maximus* preprint genome by the DNA Zoo team [49]. The *SphI-EcoRI* enzyme combination, which yielded relatively even and high fragment recovery across the genus was selected. We chose 300–450 bp fragment size, which has been widely used in previous studies [50, 51].

#### ddRAD data analysis

We used Stacks [52], a well-established modular pipeline, to process and analyze ddRAD sequencing data. The sequencing service provider provided demultiplexed ddRAD data. Low quality reads were removed from these 24 RAD sequencing datasets, and RAD tags were rescued (parameters: -c -q -r—renz_1 sphI—renz_2 ecoRI) using Stacks’ (v2.54) process_radtags tool. Then, we followed a reference-based approach to map paired-end ddRAD data to the chromosome-length Asian elephant draft assembly [49, 53] using the bowtie2 short read aligner [54]. Stacks’ gstacks program was used to assemble RAD loci from read-mapped BAM files and reads with mapping quality less than 20 were ignored when assembling loci (parameters:—min-mapq 20). The population program in Stacks was used to export results in Structure and Genpop formats while maximizing the number of polymorphic loci found in 80% of individuals (parameters:—structure—genepop -r 0.80—min-maf 0.05). This also provided summary statistics describing each RAD locus, such as haplotype diversity or haplotype richness at a RAD locus. A custom script designed (see code repository) calculated the length and the number of variant sites/SNPs of each RAD locus. If a variant is found in at least one sample, it counts towards a single variant site.

The Principal Component Analysis (PCA) is used to discern the underlying genetic diversity among the sampled individuals [55, 56]. Here we used Genepop format haplotype output from Stack’s *population* program for the PCA. ADEGENET [57] v2.1.4 R-package was used for PCA [58], and factoextra v1.0.7 [59] package was used for visualization.The population structure and admixture of Sri Lankan elephants was assessed using STRUCTURE v2.3.4 [60]. To speed up the execution, we used StrAuto v1.0 [61], a program used to parallelize the STRUCTURE execution. The STRUCTURE admixture model was run with a 50,000 burn-in period and 100,000 Markov chain Monte Carlo (MCMC) iterations for K = 1 to 10, with ten independent runs for each K. The Evanno method [62] implemented in STRUCTURE HARVESTER [63] was used to determine the potential K value. Across 10 independent runs, the mean and standard deviation of log likelihood of each K (LnP(K)) were calculated, and the Delta K [62] was derived based on the second order rate of change of mean log probability between successive K values. We used CLUMPP [64] to permute data from the 10 independent runs for each K to generate the final Q values for each individual. The results were graphically displayed using DISTRUCT [65, 70], included in the CLUMPAK software [66], which also includes the CLUMPP. In addition to the STRUCTURE Bayesian hierarchical clustering, we used ADEGENT to perform K-means clustering and a discriminant analysis of principal components (DAPC) [67], a nonparametric approach employed without any predetermined genetic model, to investigate subdivision of genetic groups.

## Results

### Mitochondrial genome assembly and divergence dating

The assembled mitochondrial genome of Sri Lankan elephant is 16,896 bp long with 38.7% of GC content. It encodes typical metazoan mtDNA genes, including 13 protein-coding genes (PCGs), 22 tRNA genes, two rRNA genes, and one non-coding control region (D-loop) (S3 Table). The gene order is identical to the *E*. *maximus* (NC_005129) [33] mitogenome.

**Fig 2:**
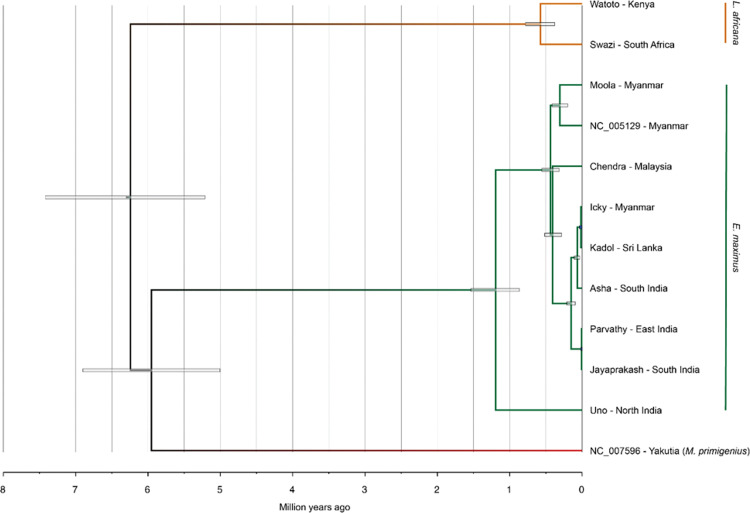
Mitochondrial phylogenetic tree of elephant samples mitochondrial reference genomes of the Asian elephant and *Mammuthus primigenius*. Node bars represent the 95% highest posterior density (HPD). Tree tips are labeled with the specimen’s name and its origin.

The mitochondrial genome of the Sri Lankan elephant was analyzed in conjunction with previously published Asian elephants, African elephants, and fossil mitogenomes (Fig 2). The fossil calibrated Bayesian analysis of the basal divergence time for all elephant mitochondrial lineages was ~6.25 million years (95% CI: 5–6.9 million years) and consistent with the previous estimates.

*Elephas-Mammuthus* clade has diverged approximately ~5.9 million years (95% CI: 5–6.9 million years) ago. The estimated coalescence time of α and β clades in Asian elephants is ~1.2 million years (95% CI: 0.75–1.5 million years). The coalescence time of the β clade is estimated at ~0.4 million years, while the Sri Lanka elephant is estimated at ~0.1 million years. *Kadol* the Sri Lankan elephant and *Icky*, the Myanmar-born elephant nested closely with elephants found in South India rather than the other India-born elephants. Moreover, Uno from North India has deeply diverged from the other Asian elephants.

**Fig 3:**
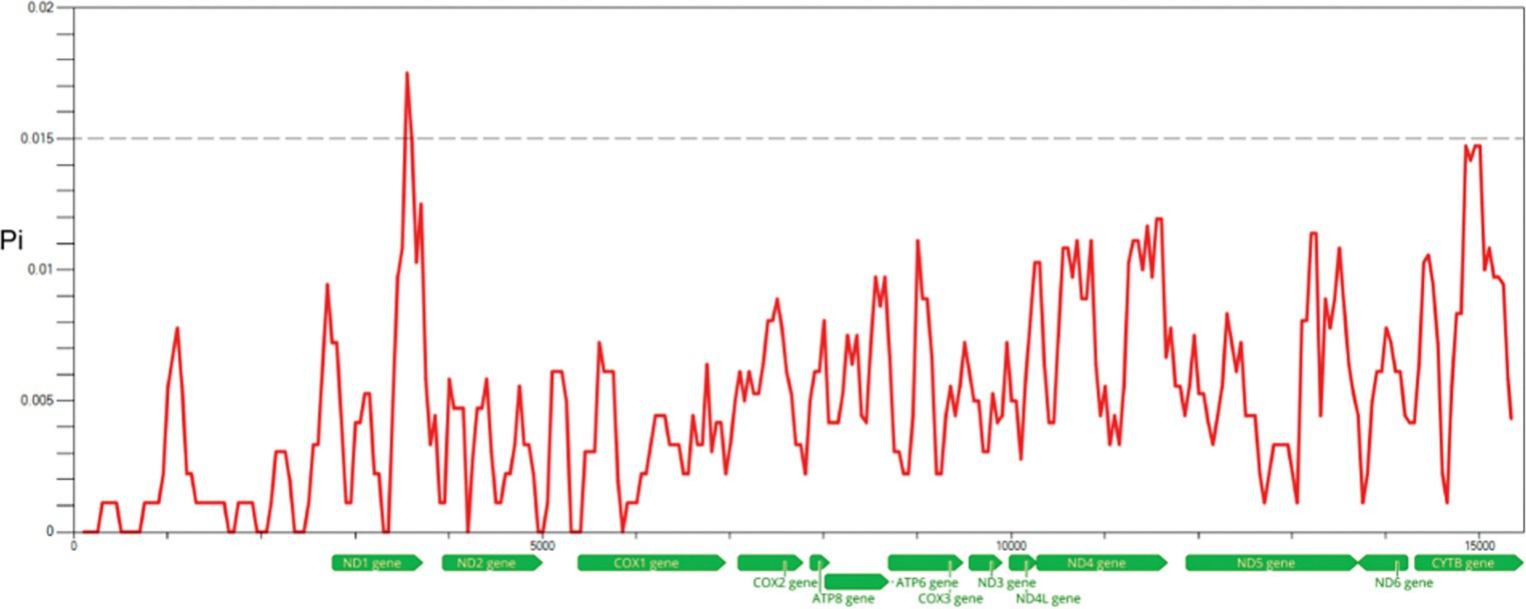
Nucleotide diversity (pi) calculated for the Asian elephant mitogenome alignment using the DnaSP program.

DNA polymorphism analysis reveals a highly varying region within the *ND1* mitochondrial gene of the Asian elephant species, and its nucleotide variability (pi) is higher than 0.015 (Fig 3). *CYTB* mitogenome also shows a higher nucleotide polymorphism than the rest of the genes in the mitochondrial genome.

### ddRAD sequencing and SNP discovery

Choosing an enzyme combination that yields relatively even and high fragment recovery across the genus is a very import step in preparing ddRAD libraries. For the fragment size selection, a narrow range will provide more consistent library recovery and would require less sequencing effort. The selected enzyme combination for this analysis, *SphI-EcoRI*, within the fragment size of 200–400 bp yielded 733M reads in total for the 24 libraries. After filtering reads with low-quality bases, a total of over 724 million high-quality paired-end reads (98.67%) were retained across 24 animals. Of those, each sample had an alignment rate of >93% to the Asian elephant reference genome (Table 2) and, the coverage ranged from 42x to 231x. Initially, we identified 409,932 loci from all samples and kept 78,950 loci (19.26%) after the quality filtering steps. Among the remaining loci, 65,600 were polymorphic. Total 51,364 SNPs were identified from the 24 samples.

**Table 2:** Read counts and alignment rates of ddRAD sequenced samples.

Sample ID	Specimen	Total reads (Millions)	Retained Reads (Millions)	Aligned reads (%)	Geographic Location
B_01	Kamani	75.87	75.4	96.49	N/A
B_02	Sandali	11.82	11.76	96.89	N/A
B_03	Uthpala	68.83	68.1	96.82	Pinnawala
B_04	Anuradhika	9.96	9.92	96.96	Pinnawala,
B_05	Kadol	11.07	11	95.74	Pinnawala
B_06	Wanamali	11.33	11.28	96.95	Pinnawala
B_08	Gangana	73.44	73.02	96.31	Pinnawala
B_09	Abaya	11.48	11.41	95.66	Kithul uthuwa
B_10	Pillu	23.63	23.55	96.12	Udawalawa
B_11	Kumari	24.82	24.74	97.66	Hambanthota
B_12	Anusha	21.82	21.76	97.08	Transferred from Dehiwala Zoo
B_13	Menika 2	23.13	23.06	96.94	Jaffna—Palali
B_14	Mathalee	10.37	10.32	96	Mathale
B_15	Meena	10.21	10.17	97.69	Meegalewa, Usgala Siyabalangamuwa
B_16	Sapumalee	11.13	11.07	96.17	Sapumalpura, settikulama
B_17	Sukumalee	62.42	62	96.89	Hambanthota origin
B_18	Maalee	60.61	60.29	96.77	Hulannuge, Siyabalanduwa
B_19	Nilgala	64.51	64.04	93.99	Nilgala,Ampaara
B_20	Migara	53.46	47.96	95.14	Ritigala
B_21	Kadira	9.76	9.71	95.57	Domestic
B_22	Parami	10.6	10.54	96.63	Domestic
B_SIN_66	Sinharaja	10.75	10.65	95.3	Sinharaja Rain Forest
B_BAN	Bandula	52.07	51.67	97.13	Dehiwala Zoo
B_MAD	Madawee	10.66	10.62	95.65	Dehiwala Zoo

Retained reads column contains the number of reads remained from the total raw reads of each sample after preprocessing with Stacks’ *process_radtags* program. Then the preprocessed datasets were aligned to the chromosome level Asian elephant reference genome, and percentages of aligned reads are contained in the final column.

### Population structure and cluster analysis

**Fig 4:**
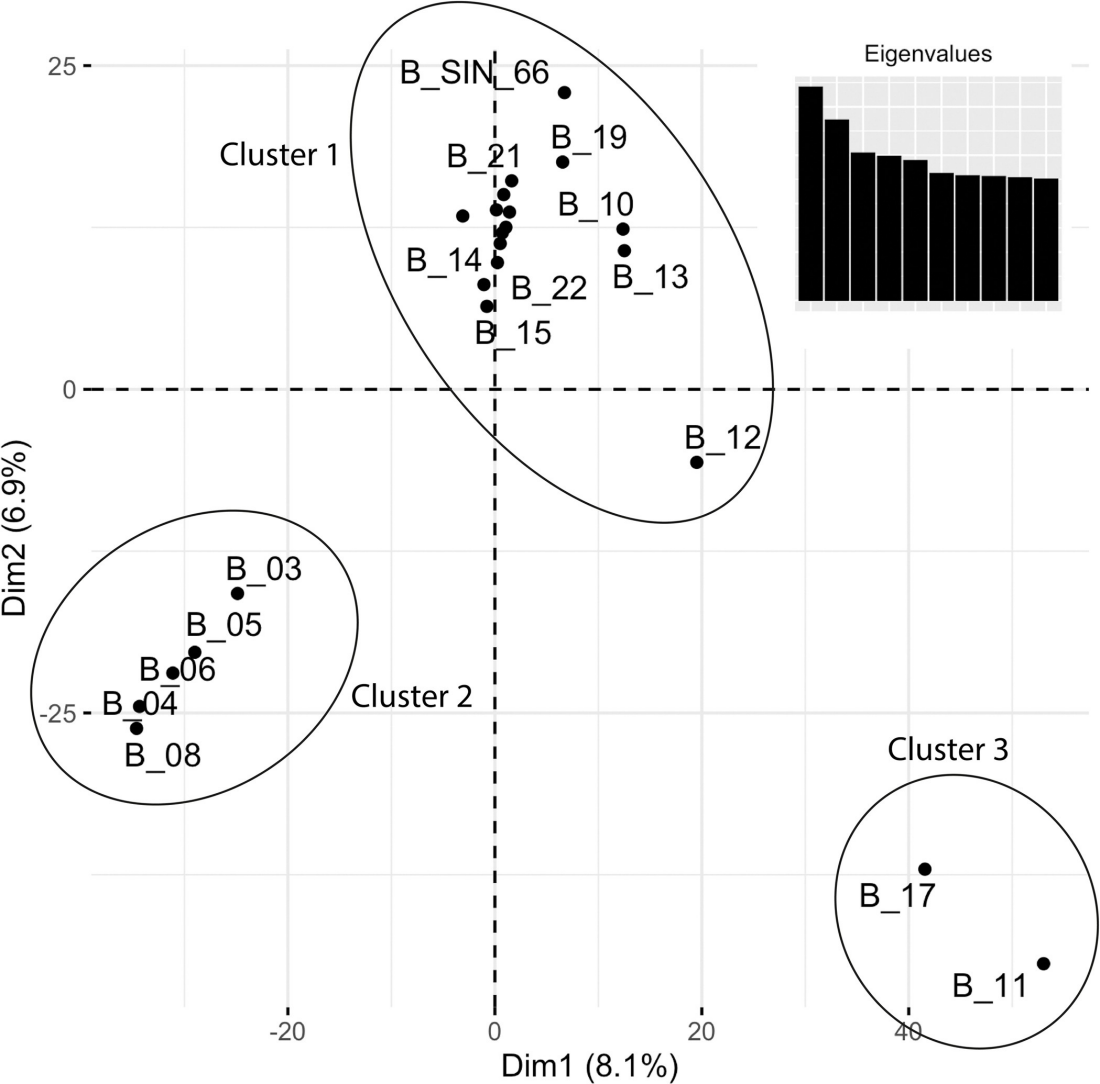
Principal component analysis (PCA) of sampled individuals. Variation explained by each principal component is depicted inside the axes brackets.

**Fig 5:**
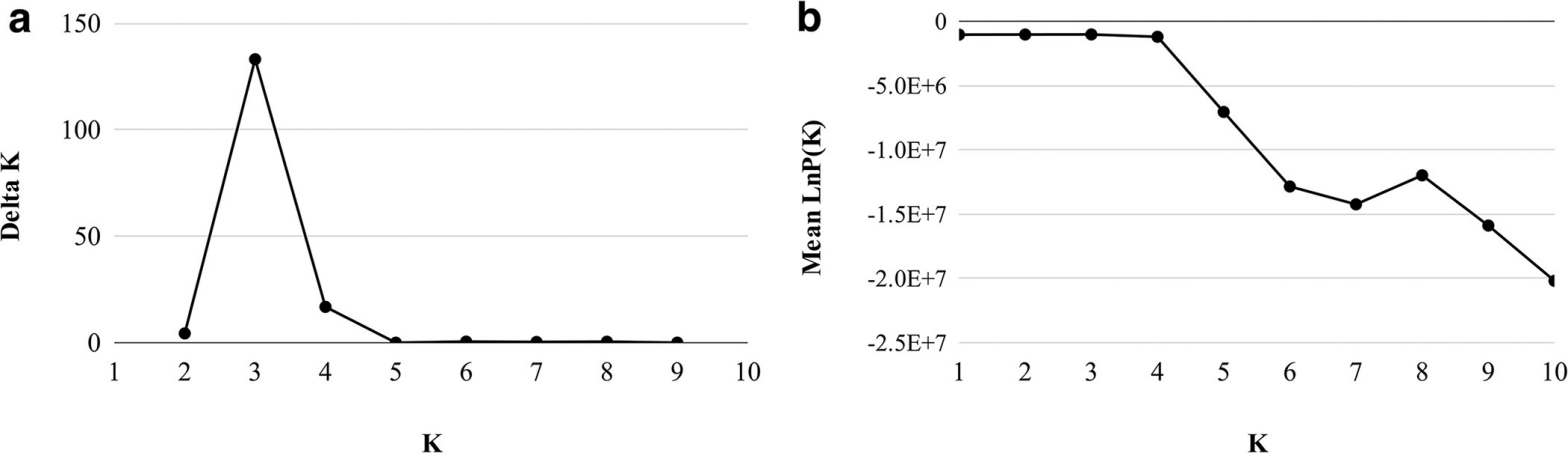
(a) LnP(K) and (b) Delta K for the structure run with 10k burn-in and 50k MCMC iterations.

The PCA analysis provided the relationships within and among populations. We performed the PCA analysis on 23 ddRAD libraries (Table 2) excluding the sample *B_MAD*, which is of African origin (Fig 5). The first and second principal components accounted for 8.1% and 6.9% of the observed variation, respectively. Three distinctive clusters (Fig 4) were identified through this analysis. The first genetic cluster included the elephants that originated in North, Northwestern, North Central, and Eastern parts of Sri Lanka, while cluster 02 comprised the elephants born in Pinnawala. Cluster 03 included the individuals born in the Southern part of Sri Lanka (S1 Table).

**Fig 6:**
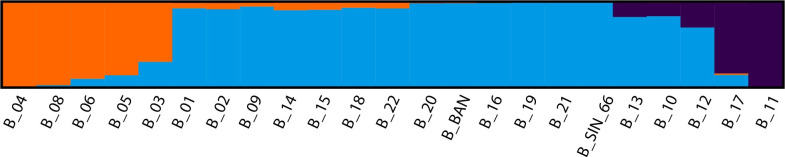
Individual assignment probabilities of 23 Sri Lankan elephants to three genetic clusters using STRUCTURE. (See S2 Fig for population structures of K ranging from 2 to 10).

Table 3 consists of the STRUCTURE HARVESTER output in the STRUCTURE analysis. According to the Evanno method, most probable K is where the Delta K attains its peak. Therefore, we can infer the optimal value for K is 3 (Fig 5A). Furthermore, the manual for STRUCTURE recommends plotting the likelihood of K for each value of K, and the point at which the plot curvature plateaus, in this study K = 3 (Fig 5B), is a potential K value. Fig 6 shows the assignment of an individual to each cluster when K = 3 and the assignments to the remaining K values are depicted in S2 Fig.

**Table 3:** STRUCTURE HARVESTER output for K = 1 to 10 with 50,000 burn-in and 100,000 MCMC iterations.

K	Reps	Mean LnP(K)	Stdev LnP(K)	Ln’(K)	|Ln’’(K)|	Delta K
1	10	-1038813.2	152.4173	NA	NA	NA
2	10	-1029847.01	626.5285	8966.19	2728.68	4.355237
3	10	-1023609.5	1409.3309	6237.51	187605.32	133.116587
4	10	-1204977.31	336841.2385	-181367.81	5665959.99	16.820862
5	10	-7052305.11	5730232.026	-5847327.8	48481.92	0.008461
6	10	-12851150.99	8735060.473	-5798845.88	4411552.81	0.50504
7	10	-14238444.06	10948785.58	-1387293.07	3642979.3	0.332729
8	10	-11982757.83	13088426.77	2255686.23	6161082.68	0.470728
9	10	-15888154.28	15345339.61	-3905396.45	395730.74	0.025788
10	10	-20189281.47	12950693.96	-4301127.19	NA	NA

**Fig 7:**
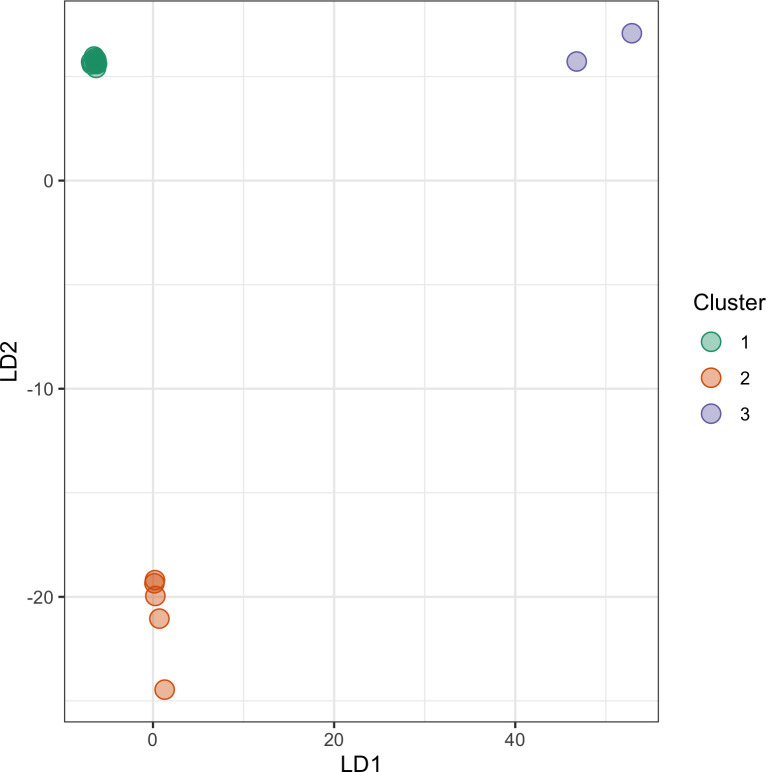
Discriminant analysis of principal components (DAPC) for ddRAD samples.

In addition to the Bayesian clustering using STRUCTURE, we performed a discriminant analysis of principal components (DAPC). DAPC is a supervised multivariate statistical method that can provide an efficient description of genetic clusters with few synthetic variables. It produces synthetic variables maximizing differences between predefined sample groups while minimizing variation within groups. Assuming three clusters exist among the samples, individuals were assigned to clusters using the K-means algorithm (Table 4). These clusters were then explained with two discriminant functions and 20 remaining PCA axes. The DAPC analysis (Fig 7) confirms the population structure inferred from STRUCTURE analysis. The cluster assignments obtained using the K-means algorithm support the grouping of elephants identified from PCA (Fig 4).

**Table 4:** Cluster assignments using K-means algorithm.

Sample ID	Specimen	Cluster 1	Cluster 2	Cluster 3
B_01	Kamani	x		
B_02	Sandali	x		
B_03	Uthpala		x	
B_04	Anuradhika		x	
B_05	Kadol		x	
B_06	Wanamali		x	
B_08	Gangana		x	
B_09	Abaya	x		
B_10	Pillu	x		
B_11	Kumari			x
B_12	Anusha	x		
B_13	Menika 2	x		
B_14	Mathalee	x		
B_15	Meena	x		
B_16	Sapumalee	x		
B_17	Sukumalee			x
B_18	Maalee	x		
B_19	Nilgala	x		
B_20	Migara	x		
B_21	Kadira	x		
B_22	Parami	x		
B_SIN_66	Sinharaja elephnat	x		
B_BAN	Bandula	x		

Cluster 1 north-eastern, Cluster 2: mid-latitude, Cluster 3:southern regions

### Haplotype diversity and SNPs counts in loci

Stack’s population program identified 67,432 assembled RAD loci with their calculated haplotype diversity and included 50,490 Genome-wide SNPs to analyze the population structure of Sri Lankan elephants. Higher haplotype diversity means its polymorphism is also higher (Table 5). The number of SNPs also shows a similar variation with the haplotype diversity. The identified regions are between 250–450bp range and could be easily amplified with PCR.

**Table 5:** Top 15 polymorphic loci sorted by the haplotype diversity.

Locus ID	Length	Scaffold	Haplotype Count	Haplotype Diversity	SNPs
371836	395	HiC_scaffold_2105	4	6.16934	13
35814	323	HiC_scaffold_3	5	5.46089	14
154646	367	HiC_scaffold_13	3	5.39403	12
379964	330	HiC_scaffold_7457	3	5.11538	12
156301	324	HiC_scaffold_13	4	5.07539	11
122662	300	HiC_scaffold_11	4	4.97582	12
150485	388	HiC_scaffold_13	5	4.69872	11
378947	378	HiC_scaffold_6623	6	4.6643	12
390538	434	HiC_scaffold_23929	6	4.2091	11
310808	287	HiC_scaffold_26	4	4.07611	9
395229	420	HiC_scaffold_46895	4	4.04979	8
96056	282	HiC_scaffold_7	2	4.00569	8
69654	279	HiC_scaffold_7	4	4.00357	10
94283/	278	HiC_scaffold_7	3	3.9704	9
398191	399	HiC_scaffold_89752	3	3.91323	8

## Discussion

Modern, historical, and ancient mitogenomic data from all three elephant species *Loxodonta africana*, *Elephas maximus* and *Mammuthus primigenius* provided insights into the historical biogeography of the elephants [16, 68]. The mitochondrial genome of the Sri Lankan tusker selected for the study was assembled *de novo*, and available references assisted in filling the gaps in the D-loop region. The phylogenetic analysis conducted to understand the evolution of Asian elephants included twelve mitochondrial genomes: two *Loxodonta*, one *Mammathus*, and nine Asian elephants, including the newly assembled Sri Lankan one. So far, the mt-D loop region and CYTB have been used to clarify the phylogeographic relationship and divergence of Asian elephants [14, 16, 69]. However, during our analysis we excluded the D-loop region because of its complexity. Our results support the deep bifurcation between African elephants, Asian elephants and Mammoths, previously been proposed based on mitochondrial genomes [39, 68, 70]. The fossil calibrated Bayesian analysis also agrees with the previous estimates [14, 71, 72] of the basal divergence time for elephant mitochondrial lineages, ~6.25 million years. *Elephas-Mammuthus* clade diverged approximately ~5.9 million years (95% CI: 5–6.9 million years) ago.

The α and β clades divergence from the Elephas is approximately 1.5 million years ago, which supports with the previous [73, 74]. The β1 sub-clade is distributed primarily in Sri Lanka, and a few populations of α clade was recorded [14, 16]. In the current study, the Sri Lankan elephant *Kadol* was nested with a Myanmar elephant named *Icky*. It was with an estimated coalescence time of ~0.1 million years, possibly due to the historical trade of elephants as gifts between Sri Lanka and Myanmar. Moreover, other South Indian elephants namely *Asha*, *Parvathy* and *Jayaprakash* has placed sister to *Kadol* and *Icky*. This further suggests the existence of β1 subclade in Sri Lanka and the Southern part of India and even Myanmar. Further, *Moola* and NC_005129 also clustered distantly with *Icky*, though all those samples are from Myanmar (β1 sub-clade). During warm periods, the Asian elephants of the β clade may have moved from Indian peninsular and Sri Lanka to the eastern and southeastern parts of Asia [13, 75]. Vidya *et al*., in 2005 [20] recorded the predominance of α clade in the Southern part of the Sri Lanka. *Kadol* from mid-latitude points out the prevalence of β1 sub-clade in the central part of Sri Lanka. However, further studies are needed with a larger number of samples to explore the coexistence of α and β clades in Sri Lanka. This coexistence of both the α and β clades in Sri Lanka and the predominance of the α clade on the mainland explained by the direction of gene flow from the larger mainland population to the smaller Sri Lankan population which occurred during more recent events of reconnection and was influenced by climate change and reflected genetic admixture of haplogroups in the Sri Lankan population [14, 16]. Although the Borneo elephants belong to β1 sub-clade [5, 12, 76], in our analysis *Chendra* distantly clustered with the other β1 main sub-clade. This genetic difference of the Bornean elephant from other mainland Asian elephant subspecies makes it one of the highest priority populations for Asian elephant conservation [7, 77]. The α clade elephants are primarily found in northern India, Nepal, across Myanmar, Thailand and Vietnam, and potentially dominant in Cambodia, while some found in Sri Lanka [5, 13]. Accordingly, the North Indian elephant, *Uno* which clustered distantly from the other Asian elephants is probably from the α clade. The male elephants disperse from herds, mediating nuclear gene flow [78–80], and female elephants are matrilocal and remain with their natal herds [81, 82]. As the mitochondrial genome is transmitted maternally, it is tied to the geographic range of the herd and sub structuring at mtDNA may be expected even in small populations [28]. Therefore, the data taking the full mitochondrial sequence (excluding D-loop) suggest the relatedness of the α and β clades. However, earlier phylogeographic and morphological analyses indicate that the Sri Lankan and Southern Indian elephants are not distinct enough to warrant classification as separate subspecies [13]. Including more genomic data from these regions would improve the phylogenetic analysis.

Since previously utilized SSR regions have specificity issues, Asian elephant-specific makers are of critical need [30]. Interestingly, the *CYTB* and *ND1* mitochondrial genes exhibit the highest nucleotide polymorphism with respect to other mitochondrial genes in our study. These two genes *CYTB* [13] and *ND1* [6] have been also used to study the population genetic structure of Asian elephants. However, mt-D-loop region has been widely used to study the elephant population discrimination [5, 13, 14, 16].

The above findings prompted us to investigate whether there is genetic diversity among elephants on the small island of Sri Lanka. Genetic diversity is the basis for evolutionary changes within a natural population. Maintaining or preserving high levels of genetic diversity is fundamental in conservation genetics [83]. Small isolated populations resulting from habitat fragmentation cause the loss of genetic diversity due to random genetic drifts and genetic bottlenecks [84]. It may limit the gene flow, causing significant consequences on the genetic diversity within isolated populations [84]. One of the best ways to achieve efficient conservation management of wild animals and to understand the phylogenetic relationships of the animals is to reveal the population structure and diversity of the animals. We employed ddRAD sequencing to examine the genetic diversity and population structure of Sri Lankan elephants from different geographical origins. Even though the total number of samples included in the analysis is low compared to the elephant population in Sri Lanka, it is a good representation. Good quality DNA is the key to the success of the ddRAD technology and obtaining blood from a large number of wild elephants is practically impossible. Therefore, we collected blood from the captive elephants having a pure wild origin from different geographical locations in Sri Lanka. The ddRAD–seq approach is an efficient and cost-effective methodology for SNP discovery. A total of 50,490 high-quality SNPs specific for Sri Lankan elephants; *E m*. *maximus* identified *w*ould be utilized for future ecological and evolutionary studies. These SNPs were evenly distributed throughout the genome, showing that ddRAD is an efficient next-generation sequencing method for genetic diversity studies. The SNPs identified can be further validated with more samples and used as an efficient base for the design of markers for the identification of Sri Lankan elephants.

Our results provide insights into elephant habitat fragmentation in Sri Lanka which may have led to different sub-populations. The genetic diversity within Sri Lankan elephants is higher, suggesting a significant differentiation at a geographical level in Sri Lanka resulting in three significant clusters; Northwest-Mahaweli, Northeast, and Southern. Habitat fragmentation may have overall positive effects on the genetic diversity of the Sri Lankan elephants. Although Fernando and colleagues [19] found the Sinharaja elephant morphologically different, genetically, it clusters with the north- eastern elephant cluster, suggesting Sinharaja population is contiguous with the dry zone population in North-east and east. The elephant population recorded in Sinharaja is limited to 3 elephants [19]. These elephants could be recent migrants to the Sinharaja rainforest, and morphological changes are adaptations to the thick rainforest. Including more samples from all identified populations will provide solid evidence for such a hypothesis. While collecting blood from wild elephants is very challenging, collecting dung is doable. Our findings indicate that the single population that has been distributed in the east-northeast and north central areas is not geographically or landscape segregated.

However, our recent work showed the challenges associated with utilizing existing SSR markers in elephant dung DNA work. As a result, using available genomic data with long reads followed by validation of a polymorphic set of SNPs would reduce the cost of such studies while producing high-quality data. The current analysis suggests possible genomic regions with a reasonable number of polymorphic SNPs. The size of the fragments is within the range of easy PCR amplification and Sanger sequencing and capillary electrophoresis techniques. Therefore, validation with more samples from Sri Lanka and other regions may lead to the identification of a unique set of SNPs for Sri Lanka elephants. The availability of such sensitive analytical methods will assist in overall conservation and management strategies when the Sri Lankan elephant population has fallen almost 65% since the 19th century.

## Conclusions

The first *de novo* assembled mitogenome presented here may further improve with long-read sequencing approaches. Our data strongly suggests a genetically closer relationship between Sri Lankan elephants to Southern Indian elephants and Myanmar elephants being in β1 clades A total of 50,490 genome-wide SNPs were identified among 24 Sri Lankan elephants, and further analysis clustered them into three clusters suggesting considerable difference. A set of polymorphic SNPs were identified for further validation. The results suggest the importance of social organization and biogeographic barriers in shaping the distribution of genetic variation among Asian elephant populations in Sri Lanka. These variables could be further assessed by including more individuals in the analysis.

## Supporting information

S1 Fig Morphology of the elephant considered for the ddRAD analysis. (A) Kamani (B) Sandali (C) Uthpala (D) Anuradha (E) Kadol (F) Wanamali (G) Gangana (H) Abaya (I) Pillu (J) Kumari (K) Anusha (L) Menika II (M) Mathalee (N) Meena (O) Sapumalee (P) Sukumalee (Q) Malee (R) Nilgala (S) Migara (T) Kadira (U) Parami (V) Sinaharaja _066 (W) Bandula (X) Madawee.
(DOCX)

S2 Fig Sri Lankan elephant population structure from K = 2 to K = 10. (DOCX)

S1 Table Descriptive data of the elephants used for the study. *No significant phenotype found.
(DOCX)

S2 Table Estimated cut sites with fragment size 200–400. (DOCX)

S3 Table Genetic contents in the mitochondria genome of *E*. *maximus maximus*. (DOCX)
