# Supplementary material for: Genetic features of Sri Lankan elephant, Elephas maximus maximus Linnaeus revealed by high throughput sequencing of mitogenome and ddRAD-seq
Source: PLoS One. 2023 Jun 13;18(6):e0285572. doi: 10.1371/journal.pone.0285572 (PMC10263358; doi:10.1371/journal.pone.0285572)
Supplement: S1 Fig — (A) Kamani (B) Sandali (C) Uthpala (D) Anuradha (E) Kadol (F) Wanamali (G) Gangana (H) Abaya (I) Pillu (J) Kumari (K) Anusha (L) Menika II (M) Mathalee (N) Meena (O) Sapumalee (P) Sukumalee (Q) Malee (R) Nilgala (S) Migara (T) Kadira (U) Parami (V) Sinaharaja _066 (W) Bandula (X) Madawee. (DOCX) [file pone.0285572.s001.docx]

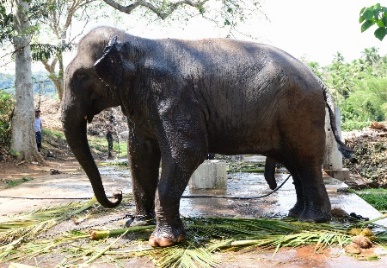

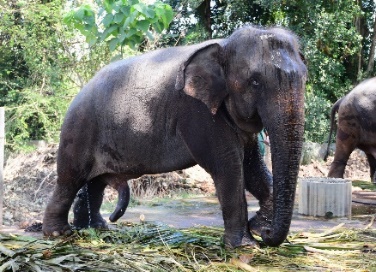

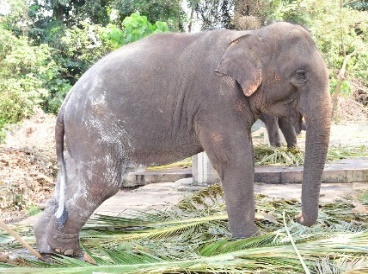

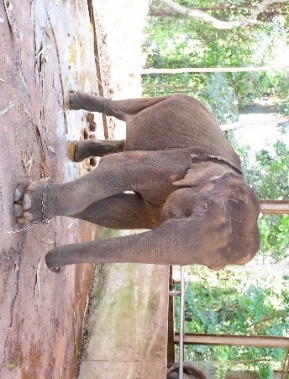

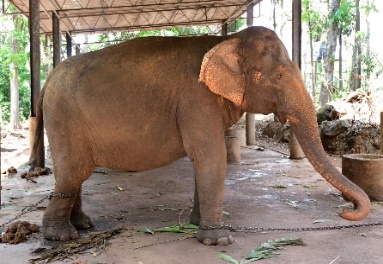

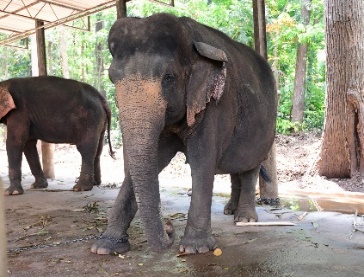

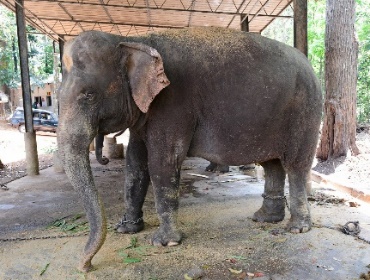

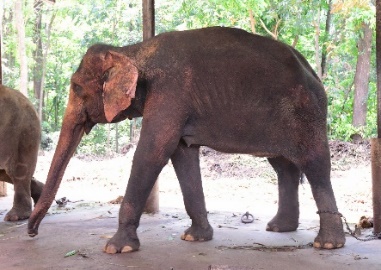

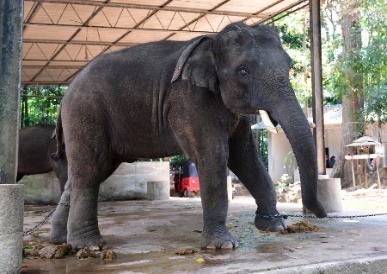

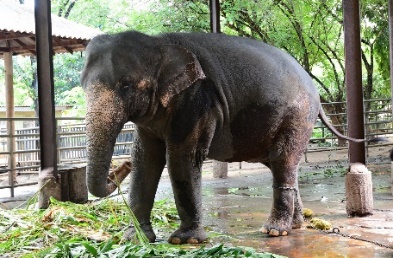

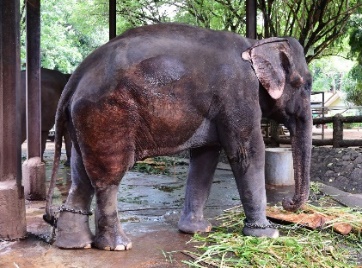

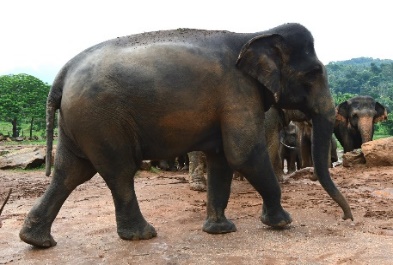

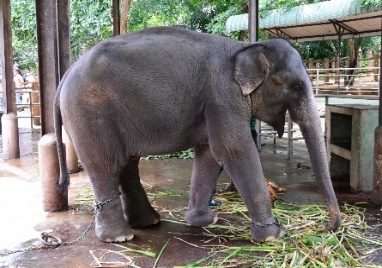

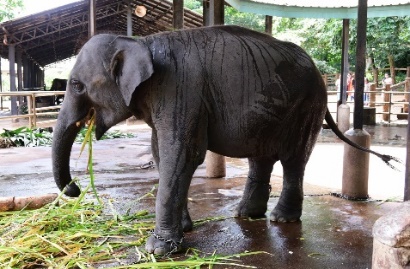

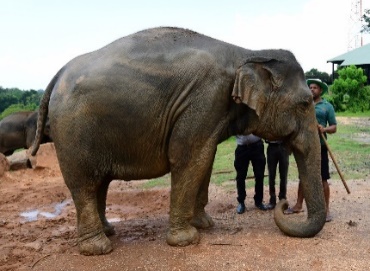

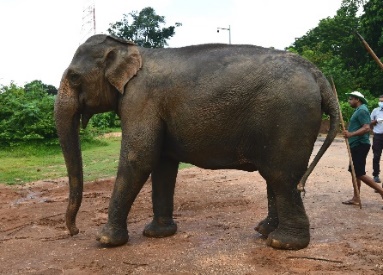

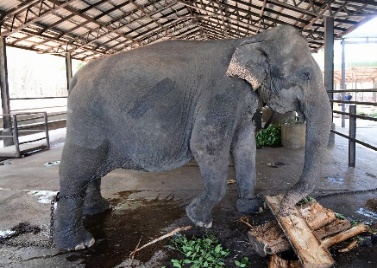

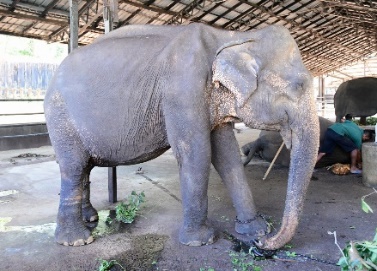

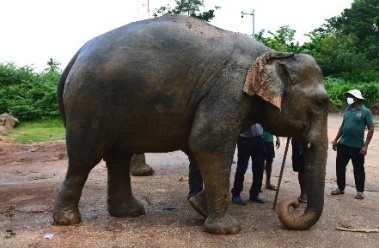

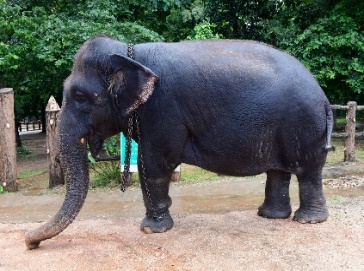

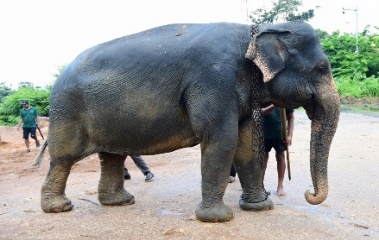

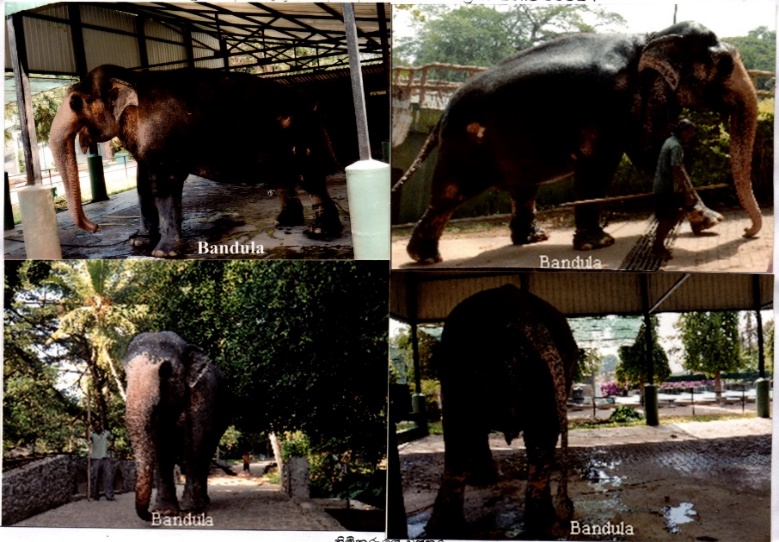

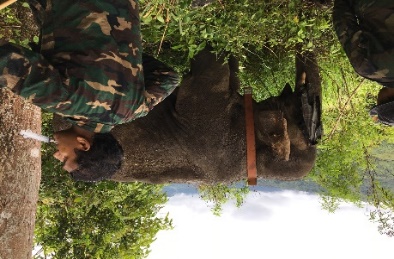

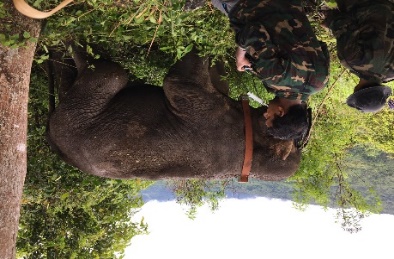

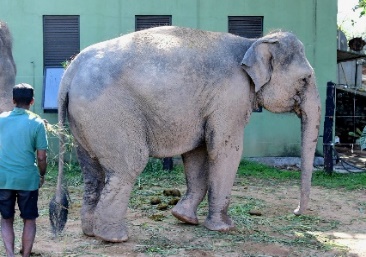


**X**

**W**

**V**

**U**

**T**

**M**

**Q**

**O**

**N**

**S**

**R**

**G**

**F**

**E**

**I**

**H**

**L**

**K**

**J**

**P**

**B**

**D**

**C**

**A**

**S1 Fig:** : Morphology of the elephant considered for the ddRAD analysis (A) Kamani (B) Sandali (C) Uthpala (D) Anuradha € Kadol (F) Wanamali (G) Gangana (H) Abaya (I) Pillu (J) Kumari (K) Anusha (L) Menika II (M) Mathalee (N) Meena (O) Sapumalee (P) Sukumalee (Q) Malee (R) Nilgala (S) Migara (T) Kadira (U)Parami (V) Sinaharaja _066 (W) Bandula (X) Madawee
