# Supplementary figures and images for: Genetic features of Sri Lankan elephant, Elephas maximus maximus Linnaeus revealed by high throughput sequencing of mitogenome and ddRAD-seq
Source: PLoS One. 2023 Jun 13;18(6):e0285572. doi: 10.1371/journal.pone.0285572 (PMC10263358; doi:10.1371/journal.pone.0285572)

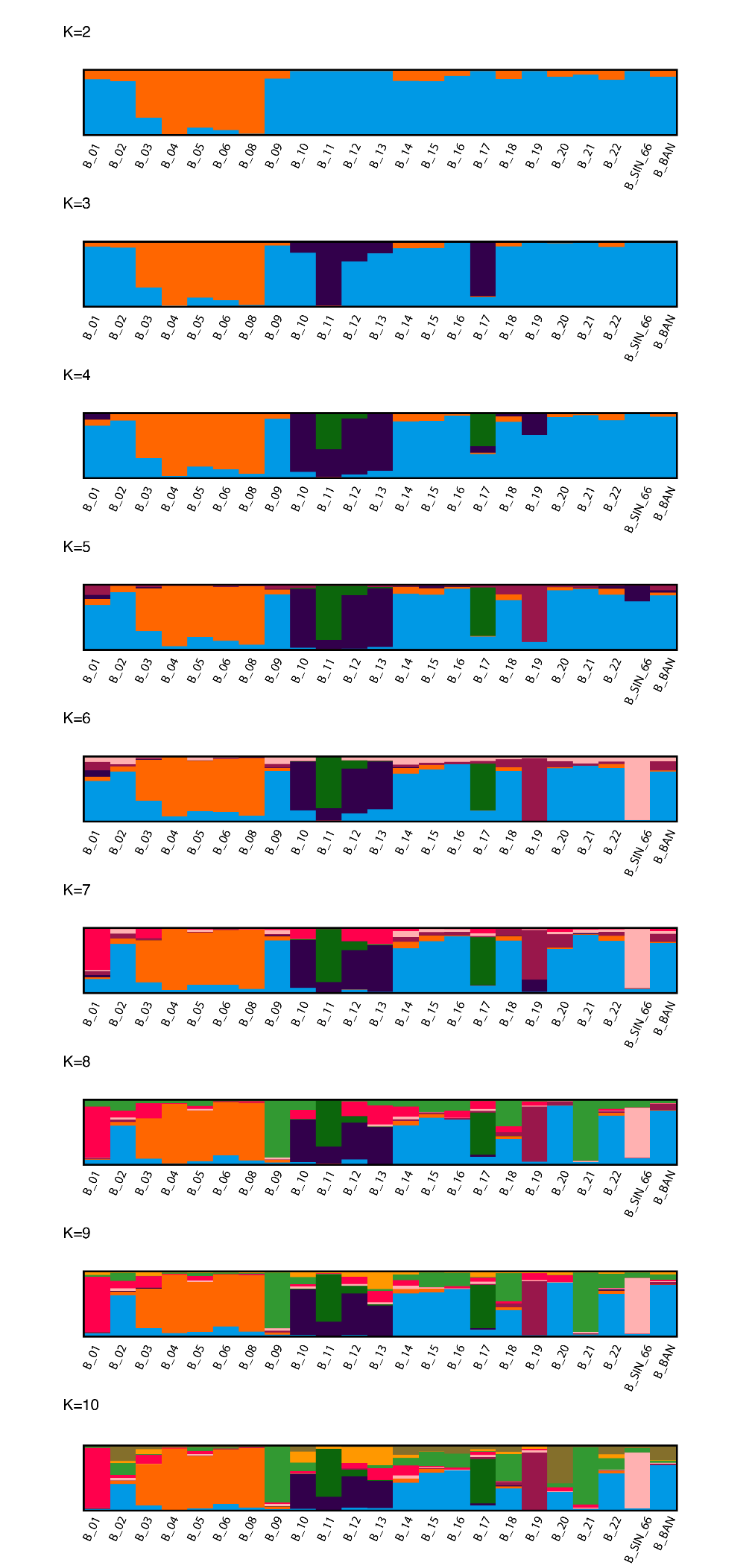


**S2 Fig :** Sri Lankan elephant population structure from K=2 to K=10

Supplement: S2 Fig — (DOCX) [file pone.0285572.s002.docx]
