# Supplementary material for: Genetic features of Sri Lankan elephant, Elephas maximus maximus Linnaeus revealed by high throughput sequencing of mitogenome and ddRAD-seq
Source: PLoS One. 2023 Jun 13;18(6):e0285572. doi: 10.1371/journal.pone.0285572 (PMC10263358; doi:10.1371/journal.pone.0285572)
Supplement: S1 Table — *No significant phenotype found. (DOCX) [file pone.0285572.s003.docx]

**S1 Table**: Descriptive data of the elephants used for the study

| **Sample ID** | **Name** | **Sex** | **Age** | **Found place** | **Morphology data** |
| --- | --- | --- | --- | --- | --- |
| B_1 | Kamani | Female | 32 | N/A | White patches present |
| B_2 | Sandali | Female | 28 | N/A | White patches present |
| B_3 | Uthpala | Female | 22 | Born in Pinnawala | White patches present |
| B_4 | Anuradhika | Female | 28 | Born in Pinnawala, North central origin | No* |
| B_5 | Kadol | Male | 10 | Born in Pinnawala, North central origin  Father – Thandula  Mother- Rejina | Tusker |
| B_6 | Wanamali | Female | 11 | Born in Pinnawala | No* |
| B_8 | Gangana | Male | 10 | Born in Pinnawala, North central origin | No* |
| B_9 | Abaya | Male | 11 | Found in kithul uthuwa | No* |
| B_10 | Pillu | Male | 13 | Transferred from Eth Athuru Sewana, Udawalawa | No* |
| B_11 | Kumari | Female | 56 | Found in Hambanthota | No* |
| B_12 | Anusha | Female | 76 | Transferred from Dehiwala Zoo | White patches present |
| B_13 | Menika 2 | Female | 31 | Jaffna - Palali | No* |
| B_14 | Mathalee | Female | 51 | Mathale | White patches present |
| B_15 | Meena | Female | 24 | Meegalewa, Usgala Siyabalangamuwa | Baby tusks, White patches present |
| B_16 | Sapumalee | Female | 24 | Sapumalpura ,settikulama | No* |
| B_17 | Sukumalee | Female | 37 | Born in Pinnawala, Hambanthota origin | No* |
| B_18 | Maalee | Female | 21 | Hulan nuge , Siyabalanduwa | No* |
| B_19 | Nilgala | Male | 9 | Nilgala,Ampaara | Baby tusks |
| B_20 | Migara | Male | 8 | Ritigala | No* |
| B_21 | Kadira | Male | 13 | court order/domestic | No* |
| B_22 | Parami | Female | 15 | court order/domestic | White patches present |
| B_SIN_66 | Sinharaja elephnat | Male |  | Sinharaja Rain forest | Tusker |
| B_BAN | Bandula | Male | 72 | Dehiwala Zoo | White patches present |
| B_MAD | Madawee | Female | 11 | Dehiwala Zoo | Short cut near the right ear, White large spot near eyes |

*No significant phenotype found
