# Supplementary material for: Genetic features of Sri Lankan elephant, Elephas maximus maximus Linnaeus revealed by high throughput sequencing of mitogenome and ddRAD-seq
Source: PLoS One. 2023 Jun 13;18(6):e0285572. doi: 10.1371/journal.pone.0285572 (PMC10263358; doi:10.1371/journal.pone.0285572)
Supplement: S2 Table — (DOCX) [file pone.0285572.s004.docx]

**S2 Table:** Estimated cut sites with fragment size 200-400

| **Enzyme combination** | **Enzyme1_seq** | **Enzyme2_seq** | **Asian(GCA_014332765.1)** | **Asian_dnazoo** | **African_loxa3** | **African_loxa4** |
| --- | --- | --- | --- | --- | --- | --- |
| EcoRI - MspI | G^AATTC | C^CGG | 76436 | 79424 | 87368 | 87395 |
| SphI - EcoRI | GCATG^C | G^AATTC | 22259 | 23101 | 23460 | 23440 |
| SphI - MluCI | GCATG^C | ^AATT | 680676 | 697908 | 731341 | 731273 |
| NlaIII - MluCI | CATG^ | ^AATT | 519197 | 529099 | 539234 | 539016 |
| SbfI - EcoRI | CCTGCA^GG | G^AATTC | 13571 | 14174 | 14575 | 14560 |
| SbfI - MluCI | CCTGCA^GG | ^AATT | 679470 | 696787 | 729964 | 730024 |
| AvaII - MspI | G^GWCC | C^CGG | 188365 | 193854 | 202257 | 202269 |
| NlaIII - MluCI | CATG^ | ^AATT | 519197 | 529099 | 539234 | 539016 |
| PstI - MspI | CTGCA^G | C^CGG | 87148 | 89998 | 98576 | 98635 |
| Mse I - Sac I. | T^TAA | GAGCT^C | 643122 | 656514 | 660526 | 660566 |
| EcoRI - BglII | G^AATTC | A^GATCT | 38696 | 40119 | 42037 | 42037 |
| PstI - EcoRI | CTGCA^G | G^AATTC | 52282 | 53670 | 53870 | 53740 |
| SalI - PstI | G^TCGAC | CTGCA^G | 24445 | 25027 | 25106 | 25112 |
| EcoRI - HindIII | G^AATTC | A^AGCTT | 41650 | 42949 | 43368 | 43368 |
| PstI - MspI | CTGCA^G | C^CGG | 87148 | 89998 | 98576 | 98635 |
| Bgl II - EcoR I. | A^GATCT | G^AATTC | 38696 | 40119 | 42037 | 42037 |
| EcoRI - BglII | G^AATTC | A^GATCT | 38696 | 40119 | 42037 | 42037 |
