# Supplementary material for: Genetic features of Sri Lankan elephant, Elephas maximus maximus Linnaeus revealed by high throughput sequencing of mitogenome and ddRAD-seq
Source: PLoS One. 2023 Jun 13;18(6):e0285572. doi: 10.1371/journal.pone.0285572 (PMC10263358; doi:10.1371/journal.pone.0285572)
Supplement: S3 Table — (DOCX) [file pone.0285572.s005.docx]

**S3 Table:**  Genetic contents in the mitochondria genome of *E. maximus maximus*

| **Functional group** | **Genes** |
| --- | --- |
| Complex I (NADH dehydrogenase subunits) | *nd1,nd2,nd3,nd4,nd4l,nd5,nd6* |
| Complex IV (Cytochrome c oxidase subunits) | *cox1, cox2, cox2* |
| Complex V (ATP synthase subunits) | *atp6, atp8* |
| Cytochrome B | *cytb* |
| Ribosomal RNAs (rRNA) | *12S rRNA, 16S rRNA* |
| Transfer RNAs (tRNA) | *trnA(ugc), trnC(gca), trnD(guc), trnE(uuc), trnF(gaa), trnG(ucc), trnH(gug), trnI(gau), trnK(uuu), trnL(uaa), trnL(uag), trnM(cau), trnN(guu), trnP(ugg), trnQ(uug), trnR(ucg), trnS(gcu), trnS(uga), trnT(ugu), trnV(uac), trnW(uca), trnY(gua)* |

The total length of all protein-coding genes in the mitochondrial genome is 11,430bp (i.e., 67.6% of the assembled mitochondrial genome). The base composition is 33.0% of A, 25.3% of C, 13.4% of G, and 28.4% of T. The 16S rRNA and 12S rRNA of the rRNA gene are 1,566bp and 961bp, respectively. The length of 22 tRNA ranges from 69 bp (tRNA-S) to 75 bp (tRNA-L), while the control region of 1,479bp is located between tRNA-P and tRNA-F.
